# Pressure and temperature effects on deep‐sea hydrocarbon‐degrading microbial communities in subarctic sediments

**DOI:** 10.1002/mbo3.768

**Published:** 2018-11-16

**Authors:** Luis J. Perez Calderon, Evangelia Gontikaki, Lloyd D. Potts, Sophie Shaw, Alejandro Gallego, James A. Anderson, Ursula Witte

**Affiliations:** ^1^ Institute of Biological and Environmental Science University of Aberdeen Aberdeen UK; ^2^ Surface Chemistry and Catalysis Group, Materials and Chemical Engineering, School of Engineering University of Aberdeen Aberdeen UK; ^3^ Marine Laboratory Aberdeen Marine Scotland Science Aberdeen UK; ^4^ Centre for Genome Enabled Biology and Medicine University of Aberdeen Aberdeen UK

**Keywords:** bacteria, deep‐sea, hydrocarbon contamination, hydrostatic pressure, sediment, temperature

## Abstract

The Hatton–Rockall Basin (North‐East Atlantic) is an area with potential for deep‐sea (2,900 m) hydrocarbon exploration. Following the Deepwater Horizon oil spill, many investigations into the responses of sediment microbial communities to oil pollution have been undertaken. However, hydrostatic pressure is a parameter that is often omitted due to the technical difficulties associated with conducting experiments at high pressure (>10 MPa). In this study, sediments from 2,900 m in the Hatton–Rockall Basin, following a one‐week decompression period in a temperature‐controlled room at 5°C, were incubated in factorial combinations of 0.1 and 30 MPa, 5 and 20°C, and contamination with a hydrocarbon mixture or uncontaminated controls to evaluate the effect of these environmental variables on the bacterial community composition. Our results revealed varying effects of pressure, temperature, and oil contamination on the composition of the bacterial community within the sediment. Temperature was the strongest determinant of differences in the bacterial community structure between samples followed by pressure. Oil contamination did not exert a strong change in the sediment bacterial community structure when pressure and temperature conditions were held at in situ levels (30 MPa and 5°C). The γ‐proteobacteria *Pseudomonas* and *Colwellia,* and several Bacteroidetes dominated communities at 30 MPa. In contrast, hydrocarbon degraders such as *Halomonas*,* Alcanivorax,* and *Marinobacter* decreased in relative abundance at the same pressure. This study highlights the importance of considering hydrostatic pressure in ex situ investigations into hydrocarbon‐degrading deepwater microbial communities.

## 1 INTRODUCTION

The increase in global energy demand has led the petroleum industry to explore deeper and more challenging oil and gas reservoirs in the marine environment. Nevertheless, present knowledge on the effect of pressure (P, hereafter) on natural hydrocarbon‐degrading microbes is limited (Joye et al., 2016). P perturbs biological structures (e.g., membranes and proteins) and processes (e.g., protein synthesis and DNA replication), and varying degrees of adaptation to P among deep‐sea organisms are widespread (Bartlett, 2002; Somero, 1992). Microbial activity related to organic matter degradation depends on the origin of the communities: Deep‐sea microbial communities adapted to high P and low temperature (T, hereafter) show higher metabolic activity at in situ environmental conditions (Tamburini, Garcin, & Bianchi, 2003; Tamburini, Garcin, Ragot, & Bianchi, 2002), while surface particle‐associated prokaryotes exhibit decreased rates of organic matter degradation with sinking through the water column (Grossart & Gust, 2009; Tamburini et al., 2006, 2009). The effect of P on hydrocarbon degradation specifically has been studied most commonly in monocultures of isolated bacteria (Grossi et al., 2010; Scoma et al., 2016) and seems to depend on the degree of adaptation to P by the individual microorganisms. For example, increasing P negatively affected the growth yield of the hydrocarbonoclastic genera *Alcanivorax* (Scoma et al., 2016) but did not influence the growth or rate of hydrocarbon degradation of a hydrocarbonoclastic and piezotolerant *Marinobacter hydrocarbonoclasticus* strain isolated from deepwaters (Grossi et al., 2010). Investigations into the effects of pressure on mixed communities are limited and have involved cultures as opposed to natural samples, reporting slower *n*‐hexadecane degradation at 50 MPa compared to 0.1 MPa (Schwarz, Walder, & Colwell, 1975; Schwarz, Walker, & Colwell, 1974). More recently, Fasca et al. (2017) demonstrated the restructuring effects of both pressure and temperature on natural surface water (0–10 m) bacterial communities from hydrocarbon production areas in Brazil. Similarly, Marietou et al. (2018) revealed decreased microbial growth and oil degradation as pressure increased in samples from deep Gulf of Mexico (GoM, hereafter) seawater, near the Macondo well.

T plays a fundamental role in regulating the activity and growth of microorganisms (Apple, Giorgio, & Kemp, 2006; López‐Urrutia, San Martin, Harris, & Irigoien, 2006). Oil degradation generally proceeds faster at mesophilic T. However, low T‐adapted microbial communities are capable of rapid oil degradation (Coulon, McKew, Mark Osborn, McGenity, & Timmis, 2007; Ferguson, Gontikaki, Anderson, & Witte, 2017; Techtmann et al., 2017; Venosa & Holder, 2007). In some cases, the degradation of polycyclic aromatic hydrocarbons by deepwater microbial communities has been reported to be higher at low T (4–5°C) compared to high T incubations (20–24°C) (Campo, Venosa, & Suidan, 2013; Liu, Bacosa, & Liu, 2017). The T dependence of cellular processes can vary greatly and may be confounded by other environmental parameters, such as hydrostatic P (Fumiyoshi, 2007; López‐Urrutia et al., 2006). However, the complex interaction between T and P on oil degradation has rarely been addressed (Fasca et al., 2017; Schwarz et al., 1975, 1974).

This study aimed to investigate the combined effect of P, T, and response to oil contamination of the bacterial community in deep sediments from the Hatton–Rockall Basin. The Hatton–Rockall Basin, west of the United Kingdom, is a deepwater region showing promise for oil and gas exploration and production (Johnson, Ritchie, & Gatliff, 2007). However, part of the basin is designated as a marine protected area due to the presence of deep‐sea sponge aggregations (protected under the Convention for the Protection of the Marine Environment of the North‐East Atlantic), offshore deep‐sea mud habitats, and unique geological features known as polygonal faults (Joint Nature Conservation Committee, https://jncc.defra.gov.uk/page-6482). A potential deep‐sea oil spill, such as the *Deepwater Horizon* (*DwH*) incident in the GoM, could thus have detrimental effects on sensitive ecosystems in the wider area. The intrinsic capability of natural microbial communities in the Hatton–Rockall Basin to mitigate the effects of an oil spill is currently unknown. The microseepage of oil and gas in the Hatton–Rockall Basin is not sufficient to cause the establishment of oil‐degrading microbial communities in sediments (Wenger & Isaksen, 2002), and thus, microbial communities in the area may not be preconditioned to oil degradation. To our knowledge, this is the first study to evaluate the combined effect of P and T on the structure of oil‐degrading microbial communities in deep‐sea sediments.

## 2 MATERIALS AND METHODS

### 2.1 Sediment and seawater collection

Sediment and seawater samples were collected during the DY0521 cruise onboard *RRS Discovery* on May 2016 from the Hatton–Rockall Basin at 2,900 m deep (54°40.0′N, 12°16.0′W, Supporting Information Figure S1). Sediment was collected using a mega‐corer (Osil, UK) fitted with acrylic cores (internal diameter = 10 cm, length = 60 cm). The top 2.5 cm of sediment from 16 cores was sliced off, combined, and stored with aeration in a T‐controlled room at 2–4°C for one week. Upon arrival to the laboratory, sediments and seawater were stored aerated in a T‐controlled room at 5°C for 1 week prior to the initiation of the experiment. Seawater was collected from the same station above the seabed using a CTD rosette equipped with Niskin bottles.

### 2.2 Experimental design

Polyethylene incubation pouches (10 × 20 cm) were filled with 51 g of wet sediment and 20 ml of seawater, which were thoroughly mixed with 1.2% vol/vol of a mixture of hydrocarbons (model oil, hereafter). Model oil contained 21 hydrocarbons and was formulated with a mixture of aliphatic, monoaromatic, polyaromatic (PAH), and resin components (Supporting Information Table S1) with a density of 0.88 mg/cm, equivalent to a medium crude oil. The preparation procedure of model oil has been described elsewhere (Ferguson et al., 2017). The model oil was filter‐sterilized through a 0.22‐μm Teflon filter (VWR) prior to addition to sediments. The incubation pouches were heat‐sealed at the top and placed in a secondary pouch and heat‐sealed again to avoid fluid loss and minimize the contamination risk. The pouches were placed and incubated for six months at two Ps (0.1 and 30 MPa) and two Ts (5 and 20°C), in triplicate, in cylindrical pressure core‐shaped chambers (internal length = 500 mm, internal diameter = 90 mm, pressurized with a manual lever water pump to 30 MPa) and polyethylene boxes for samples at 30 and 0.1 MPa, respectively. Jackson, Witte, Chalmers, Anders, and Parkes (2017) provide a detailed description of the pressure chamber design. At the end of the incubation, the entire content of each pouch was emptied into 125‐ml glass vials. Two replicates of 2 ml slurry samples were aseptically aliquoted into Eppendorf tubes and stored at −80°C until DNA extraction. The remaining sample was left in the vial and stored at −20°C for hydrocarbon analysis. Control samples without model oil were incubated in parallel with model oil‐containing incubations. Ps and Ts were chosen to represent in situ (30 MPa and 5°C) and ambient (0.1 MPa and 20°C) P‐T conditions. Note from this that the sediments were not supplied with oxygen or nutrients for the duration of the experiment and the only nutrient sources provided for the microbial communities were found in the natural sediment and seawater, and hydrocarbons supplied at the initiation of the experiment.

Background sediment samples were processed in triplicate to determine particle size distribution and carbon content. The procedures for particle size distribution and carbon content measurements have been described elsewhere (Perez Calderon et al., 2018). The sediment was silt dominated (83.0% ± 2.2%) and had low organic carbon content (0.47% ± 0.23%) (Supporting Information Table S2).

### 2.3 DNA extraction, sequencing, and analysis

Total genomic DNA in slurry samples was extracted using the FastDNA™ SPIN Kit for Soil and the FastPrep® Instrument according to manufacturer's instructions (MP Biomedicals, Santa Ana, CA). The V3–V4 region of the 16S rRNA gene was amplified using 2× KAPA HiFi HotStart ReadyMix (Roche Diagnostics, UK) with 16S amplicon PCR forward and reverse primers as described in the 16S sample preparation guide by Illumina (https://support.illumina.com/). Amplification was performed on a Techne thermal cycler as follows: initial denaturation at 95°C for 3 min followed by 25 cycles of 30 s at 95°C, 30 s annealing at 55°C, 30 s primer extension at 72°C, and a final extension at 72°C for 5 min. Agarose gel electrophoresis (1.2% agarose, 80 V, 45 min) verified the success of PCR amplification based on the predicted amplicon size (~550 bp). PCR products were then prepared for sequencing using the Nextera XT DNA Library Preparation Kit (Illumina, San Diego, California, USA) by the Centre for Genome Enabled Biology and Medicine (University of Aberdeen), and sequenced using the Illumina MiSeq sequencer using Illumina V3 chemistry producing 300 bp paired‐end reads. Between 40,773 and 290,871 reads were sequenced per sample. One sample (Control treatment, 20°C, 0.1 MPa) was not sequenced due to the lack of sufficient PCR product.

### 2.4 Hydrocarbon extraction and analysis

Sediment subsamples of 5 g were spiked with surrogate standards prior to extraction (100 µl of pristane and toluene in dichloromethane at 20 µl/ml each), and then hydrocarbons were extracted by sonication in 20 ml of dichloromethane in 30‐ml glass vials for 10 min. Subsequently, extracts were decanted into 30‐ml glass centrifuge tubes and centrifuged for three min at 50 g to remove suspended sediment from solution. Each sample was extracted sequentially three times, and extracts were pooled in 100‐ml round bottom flasks (approximately 60 ml of extract). Thereafter, combined extracts were rotary evaporated to approximately 10 ml and transferred to a 25‐ml glass vial to be further concentrated by evaporation to approximately 1 ml under a gentle nitrogen stream. Finally, extracts were transferred to vials for analysis by gas chromatography with flame ionization detection. The configuration of the gas chromatograph is described in Ferguson et al. (2017) with the addition of 6 min holding the oven T at 300°C at the end of the ramp to ensure elution of the heavier components. In between decanting and transferring steps of the extractions, the internal walls of the vessels were rinsed with dichloromethane and the resulting solution was added to the extraction vessel.

Retention times and linearity for responses of hydrocarbons in gas chromatography analysis were determined by 6‐point calibration curves. Method blank extractions of uncontaminated sediment were carried out to ensure the sediment did not contain any of the hydrocarbons used in the experiment, and laboratory control extractions with known hydrocarbon concentration were carried out to ensure there was no preferential extraction of hydrocarbons. Blank hydrocarbon extractions were carried out with every batch of samples to ensure there was no sample cross‐contamination. Samples in which internal standard recovery (toluene for BTEX components and pristane for the other components) was less than 50% were removed from the dataset.

### 2.5 Bioinformatic and statistical analysis

Bioinformatic analysis of the amplicon sequencing data was carried out using Mothur (version 1.39.0) (Schloss et al., 2009) with alignment and taxonomic assignment against the SILVA database (release 128) (Quast et al., 2012), chimera removal using UCHIME (Edgar, Haas, Clemente, Quince, & Knight, 2011), and clustering using the OptiClust algorithm (Westcott & Schloss, 2017) at 97% similarity. Subsampling to 12,255 OTU counts per sample was applied. This leads to the exclusion of one sample (Oil treatment, 20°C, 30 MPa) from all subsequent analysis as it fell below the subsampling threshold at 4,577 sequences. Following singleton removal, 5,274 operational taxonomic units (OTUs) were identified across the 22 samples. As singleton removal leads to differing numbers of sequences per sample, subsampling was repeated at 11,746 sequences per sample for diversity analyses. Rarefaction curves showed that the number of observed samples was only moderately increasing at the subsampling depth of 11,746 (Supporting Information Figure S2). Alpha diversity (observed OTUs and Shannon index (Shannon, 1948)) and beta diversity (principal coordinate analysis using Bray–Curtis distance measure (Bray & Curtis, 1957)) metrics were calculated using Mothur (1.39.0) (Schloss et al., 2009) and QIIME (version 1.9.0) (Caporaso et al., 2011) and visualized using R (version 3.3.2) (R Development Core Team, 2017). Values for rarefaction curves were produced using the observed species values with Mothur and visualized using R. Phylotype files at phylum, family, and genus levels were created using QIIME and were visualized using R.

Statistical testing of the taxonomic content stratification of samples by metadata category was performed using analysis of molecular variance (Schloss & Handelsman, 2006), implemented in Mothur. To detect taxa with significantly different abundances between samples types, the OTU table was converted to a PhyloSeq object (version 1.19.1) (McMurdie & Holmes, 2013) and differential abundance testing was carried out using edgeR (version 3.16.5) (Robinson, McCarthy, & Smyth, 2010).

Effects of T and P on hydrocarbon recovery were modeled for individual hydrocarbons using analysis of variance in R. The response variable was hydrocarbon recovery (percentage), and the explanatory variables (as factors) were T and P. The same procedure was applied for grouped hydrocarbons: total petroleum hydrocarbons, total aliphatics, and total PAHs. Similarly, effects of T, P, and treatment as well as all possible interactions between them on the Shannon index and relative abundance at the family level were tested using analysis of variance. Model simplification was performed by stepwise elimination of nonsignificant terms from higher to lower order terms using analysis of variance until all remaining terms were found to be significant (Crawley, 2007). Assumptions of homoscedasticity, normality, and random errors were assessed visually in R. Figures were produced using package ggplot2 (Wickham & Chang, 2009).

## 3 RESULTS

### 3.1 Microbial community composition

#### 3.1.1 Effects of temperature, pressure, and oil contamination on bacterial diversity

**Figure 1:**
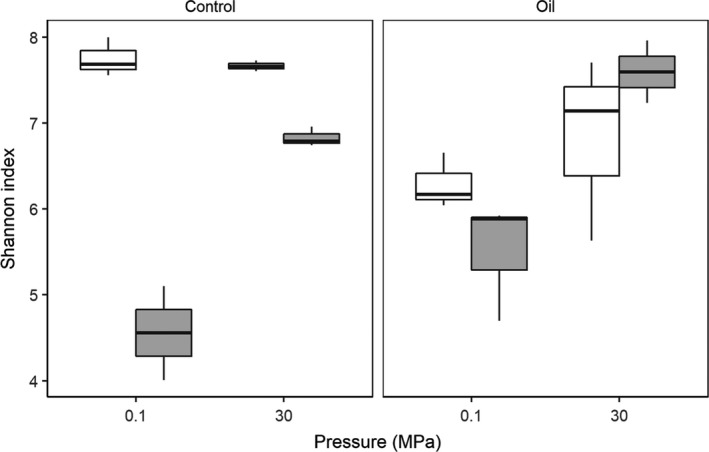
Shannon index following sediment incubations experiments. 5 (white) and 20°C (gray) incubations are shown as box plots. Atmospheric (0.1 MPa) and in situ (30 MPa) pressures are shown on the x‐axis (*n* = 3)

Alpha diversity metrics measure the richness and phylogenetic diversity of the microbial community. Stratifying samples by all three metadata categories showed that oil contamination at 5°C caused a decrease in diversity only at 0.1 MPa (Figure 1). Additionally, the effect of T on Shannon index varied with both oil contamination and P (*p* = 0.0016 and 0.0007, respectively; Supporting Information Table S3).

**Figure 2:**
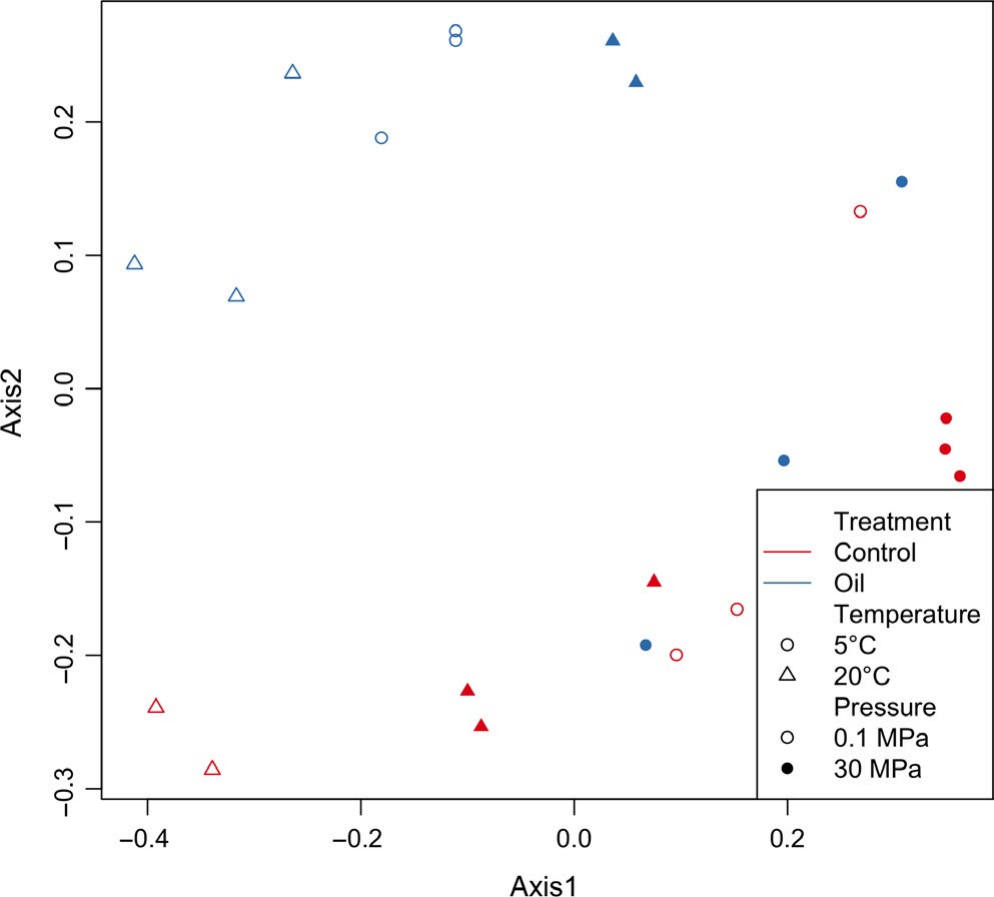
Principal coordinate analysis plot of Bray–Curtis distances split by pressure, temperature, and oil contamination in sediment incubations experiment

Beta diversity metrics measure the similarity of microbial communities based on taxa identity and abundance. Results from the Bray–Curtis metric showed a clear stratification of samples based on all three metadata categories and that, in most cases, replicates of the same condition clustered together (Figure 2). This was supported by analysis of molecular variance, which showed significance for oil contamination, T and P (*p* = 0.008, 0.003 and <0.001, respectively).

#### 3.1.2 Effects of temperature, pressure, and oil contamination on taxonomic composition

**Figure 3:**
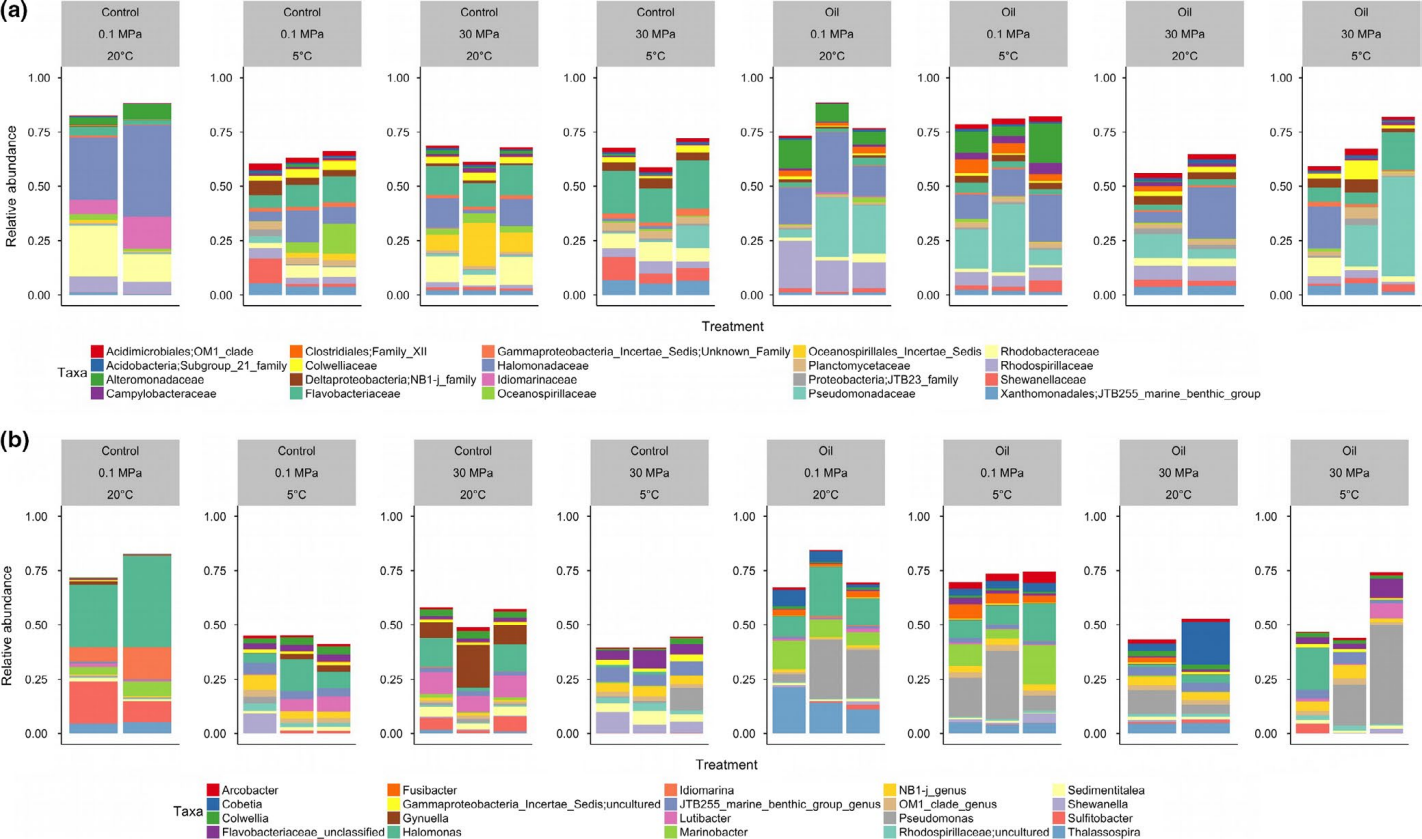
Relative abundance of the 20 most abundant taxa at the family (a) and genus (b) level stratified by oil contamination (control or oil), pressure (0.1 or 30 MPa), and temperature (5 or 20°C) from top to bottom

**Figure 4:**
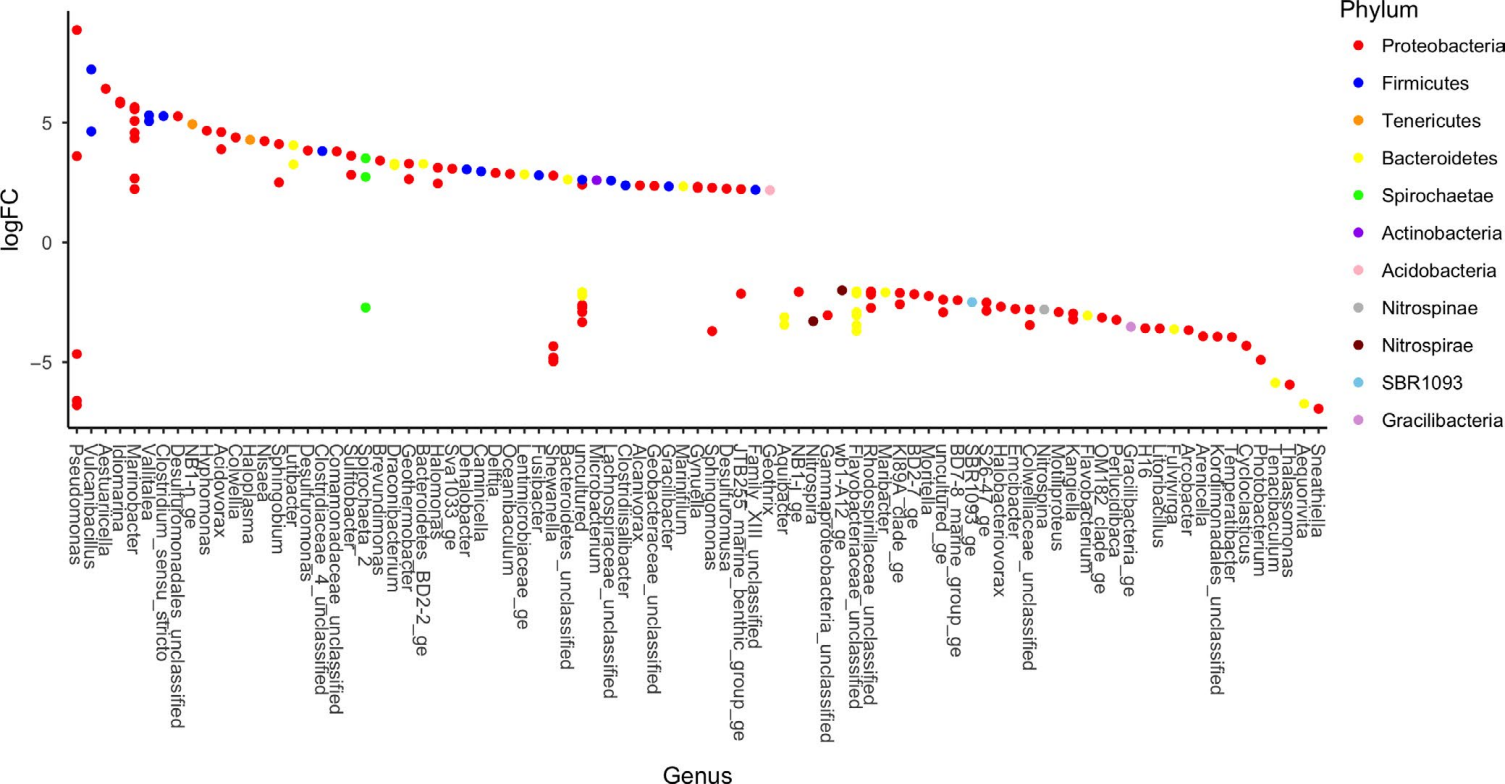
Log fold change of taxa with significantly different abundances between 5 and 20°C samples at the genus level, colored by phylum (adjusted *p* < 0.05). Positive logFC represents higher expression in 20°C incubations, and negative logFC represents higher expression in 5°C incubations

**Figure 5:**
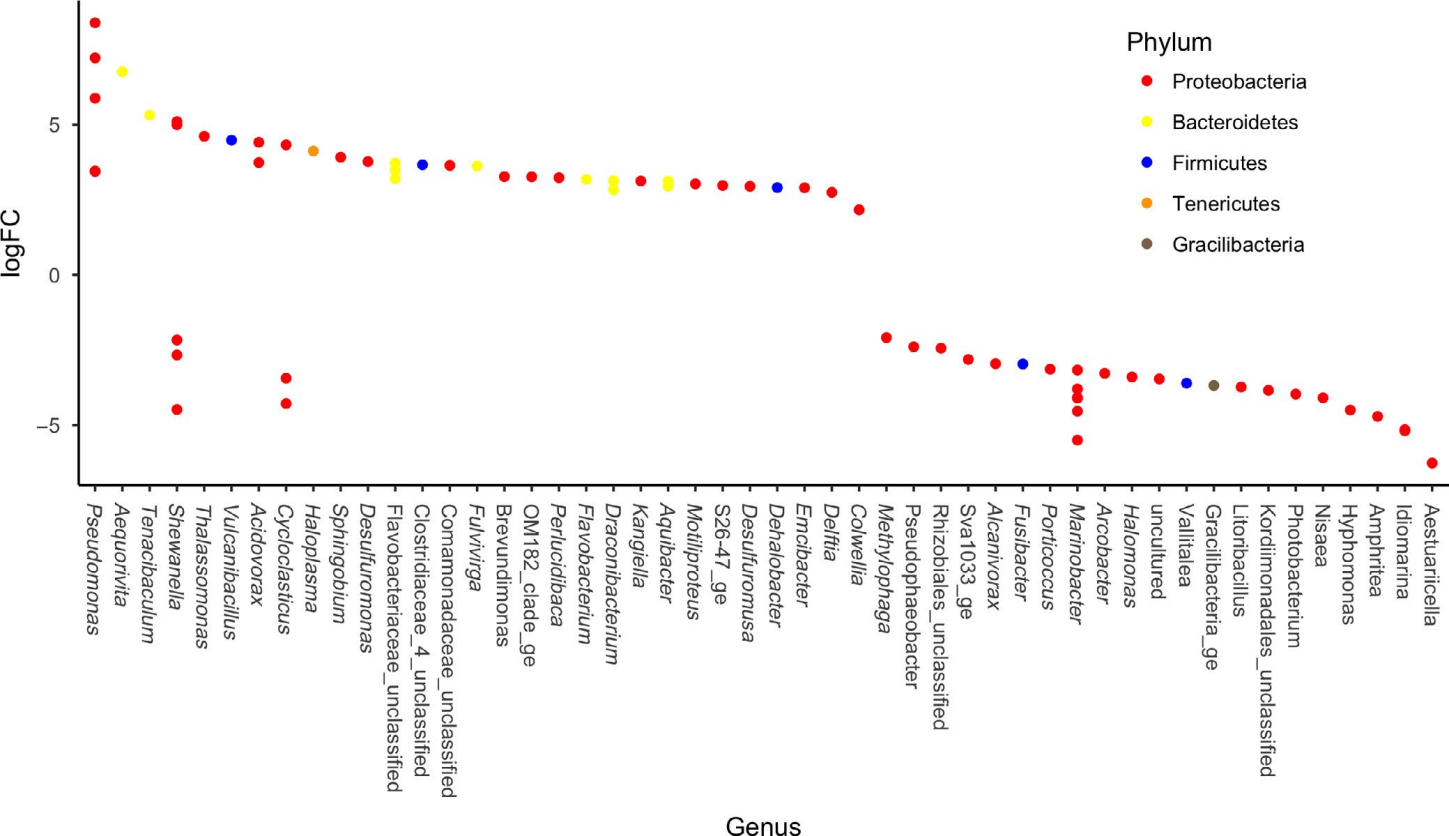
Log fold change of taxa with significantly different abundances between 0.1 and 30 MPa samples at the genus level, colored by phylum (adjusted *p* < 0.05). Positive logFC represents higher expression in 30 MPa incubations, and negative logFC represents higher expression in 0.1 MPa incubations

**Figure 6:**
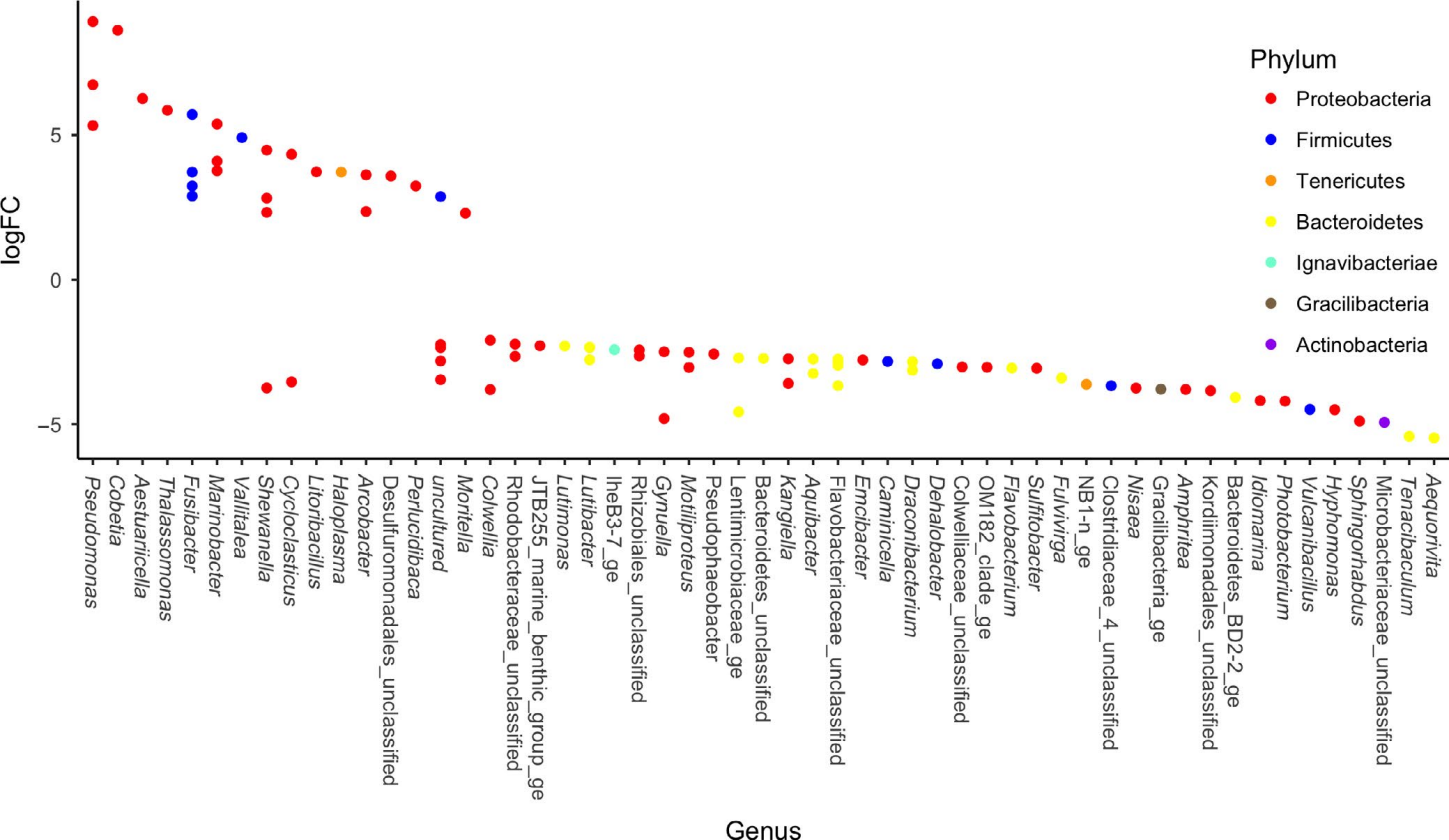
Log fold change of taxa with significantly different abundances between control and oil‐contaminated incubations at the level of genus, colored by phylum (adjusted *p* < 0.05). Positive logFC represents higher expression in oil samples, and negative logFC represents higher expression on control incubations

Summarizing the relative abundance of each taxon at the phylum level showed that overall communities were similar across all samples (Supporting Information Figure S3). The community was dominated by γ‐proteobacteria across all conditions tested. There was irregular presence of Firmicutes across samples with higher abundance in oil‐contaminated samples and 20°C than in other variable combinations (Supporting Information Figure S3). A Firmicutes *Fusibacter* OTU was found in significantly higher abundance in 0.1 MPa, 20°C, and oil‐contaminated incubations (Figures 3, 4, 5, 6).

At the family level, there were distinct differences in community composition across the variables tested (Figure 3, Supporting Information Table S4). Flavobacteriaceae (Bacteroidetes) were present in significantly higher relative abundance at 30 MPa than at 0.1 MPa (*p* = 0.0002). In contrast, Alteromonadaceae were present in significantly higher relative abundance at 0.1 MPa than at 30 MPa (*p* = 0.0003). Oil contamination resulted in an increase in relative abundance of Alteromonadaceae and Pseudomonadaceae (*p* = 0.0053 and 0.0018, respectively), and a decrease in relative abundance of Flavobacteriaceae, Rhodobacteraceae, and Alcanivoraceae (*p* = 0.0001, 0.0006, and 0.0003, respectively).

A detailed analysis to detect significantly different taxa abundances at the genus level between samples was performed using edgeR. A total of 185 taxa were found to have significantly different abundances when comparing samples at 5 and 20°C (adjusted *p* < 0.05; Figure 4). Seventy‐two taxa were found to have significantly different abundances (adjusted *p* < 0.05; Figure 5) when comparing samples held at 0.1 and 30 MPa, and 88 taxa were significantly different between control and oil‐contaminated samples (adjusted *p* < 0.05, Figure 6).

Here, the focus is placed on changes in the relative abundance of obligate hydrocarbon‐degrading bacteria (HDB) and of heterotrophic bacterial taxa often associated with petroleum contamination (Figures 3, 4, 5, 6 and Supporting Information Tables S5–S7). In situ T (5°C) resulted in γ‐proteobacteria genera *Thalassomonas, Shewanella, Moritella, Cycloclasticus,* and two unclassified Colwelliaceae OTUs to be present in higher relative abundance (Figure 4 and Supporting Information Table S5). On the other hand, 20°C incubations resulted in increased relative abundance of γ‐proteobacteria genera *Alcanivorax, Halomonas, Marinobacter*,* Colwellia*,* Idiomarina, Aestuariicella,* and the α‐proteobacterium *Sulfitobacter*. Bacteroidetes *Aequorivita*,* Flavobacterium, Tenacibaculum, Fulvivirga,* and *Aquibacter* OTUs were found in significantly more relative abundance in 5°C than in 20°C incubations.

Changes in P led to distinct shifts in microbial community composition (Figure 5 and Supporting Information Table S6). The HDB that were present in significantly higher relative abundance at 30 MPa were *Pseudomonas*,* Thalassomonas,* and *Colwellia*, and the Bacteroidetes *Aequorivita*,* Flavobacterium, Tenacibaculum, Fulvivirga,* and *Aquibacter*. In most cases, the OTUs that were favored at 20°C (*Alcanivorax, Halomonas, Marinobacter*,* Idiomarina, Aestuariicella,* and *Sulfitobacter*) were also present in higher relative abundance at 0.1 MPa, except for *Sulfitobacter*, which did not show P sensitivity. The *Amphritea* (Oceanospirillales) and the α‐proteobacterium *Pseudophaeobacter* OTUs had higher relative abundance at 0.1 MPa than 30 MPa. A *Cycloclasticus* OTU selected for at 5°C incubations was also present in significantly higher relative abundance at 30 MPa, while two other *Cycloclasticus* OTUs were selected for at 0.1 MPa.

Among the γ‐proteobacteria previously identified as capable of consuming hydrocarbons (either obligate hydrocarbonoclastic or heterotrophic), *Pseudomonas*,* Cobetia*,* Thalassomonas*,* Marinobacter, Moritella, Shewanella*, and *Aestuariicella* OTUs increased in relative abundance after oil contamination (Figure 6 and Supporting Information Table S7). In contrast, *Colwellia*,* Idiomarina*,* Tenacibacterium,* and *Sulfitobacter* OTUs were found in significantly lower relative abundance following oil contamination. Similarly, Bacteroidetes OTUs were present in significantly lower relative abundance in oil‐contaminated incubations.

### 3.2 Hydrocarbon recovery

Hydrocarbon recovery did not vary significantly with either T, P, or their interaction for any hydrocarbon or hydrocarbon group for the duration of the experiment (*p* > 0.05). Hydrocarbon recovery was highly variable across model oil components and averaged below 50% for most P‐T combinations, except eicosane (52.5% ± 5.3% at 0.1 MPa and 20°C), docosane (67.6% ± 11.0% [0.1 MPa, 20°C]; 53.0% ± 12.2% [30 MPa, 20°C]), and tetracosane in all treatment combinations (85.2% ± 62.0% [0.1 MPa, 5°C], 69.5% ± 60.4% [30 MPa, 5°C], 125.3% ± 19.8% [0.1 MPa, 20°C], 99.6% ± 25.9% [30 MPa, 20°C]) (Supporting Information Figures S4 and S5). Volatile hydrocarbons had zero or near zero recoveries (BTEX, naphthalene, and short aliphatics). In general, hydrocarbon recovery was higher at 20°C than at 0°C, regardless of P, although these differences were not significant (*p* > 0.05).

## 4 DISCUSSION

High hydrostatic P environments (defined as applying to water depths >1,000 m) account for 88% of the volume of the oceans (Jannasch & Taylor, 1984). Deep‐sea microbial communities are adapted to high hydrostatic P and exhibit higher rates of metabolic activities (hydrolytic enzymatic activities, carbon uptake, respiration, and biomass production) at in situ P compared to estimates obtained under decompressed conditions (Tamburini, Boutrif, Garel, Colwell, & Deming, 2013). Measuring the activity of deepwater microbial communities at 0.1 MPa may thus lead to an underestimation of the rates of transformations of organic carbon and may minimize current estimates of global fluxes of carbon in the ocean (Tamburini et al., 2013). Nevertheless, the effect of P on the function of oil‐degrading deepwater bacteria remains unclear. Low T and high P have been found to act synergistically to slow microbial growth and thus oil degradation at depth (Marietou et al., 2018; Schwarz et al., 1975). Indeed, several studies agree that hydrocarbon degradation at low T is characterized by a longer lag phase prior to the initiation of the degradation process (Campo et al., 2013). After 3 months of incubation however, Rodríguez‐Blanco, Antoine, Pelletier, Delille, and Ghiglione (2010) did not measure significant differences in the degradation of aliphatic hydrocarbons at 4, 10, or 25°C by seawater microbial communities from the Mediterranean Sea. Similarly, no significant differences in hydrocarbon recovery were observed between the combinations of T and P levels tested in this study after 6 months of incubation. The above observations suggest that the early stages of oil degradation are highly dependent on environmental factors; at deep‐sea conditions of T and P, the rate of oil degradation is slower due to a longer lag phase in the establishment of the oil‐degrading microbial community.

All variables (P, T, and oil contamination) had an impact on the beta diversity of the bacterial community of sediment samples. T was the strongest determinant of differences in the bacterial community structure between samples followed by P. Oil contamination had an overall significant effect on alpha diversity only in conjunction with T. At 20°C, replicates of the same conditions clustered together, that is, each P‐T‐contamination combination was characterized by a distinct bacterial community structure. On the other hand, when T was held at in situ levels (5°C), the effects of P and oil contamination were less pronounced; only oil‐contaminated samples incubated at 5°C and 0.1 MPa seem to have developed distinct and less diverse bacterial communities. This indicated that oil contamination did not exert a strong change in the sediment bacterial community structure when P and T conditions were held at in situ levels. The results here suggest that compositional changes in deep‐sea sediment bacterial communities due to oil contamination may be exaggerated in studies conducted at atmospheric P and at higher than in situ T.

The majority of γ‐proteobacteria that belong to the obligate HDB or have been associated with oil pollution in the literature were favored by atmospheric pressure and high T in this study. The genera *Halomonas, Marinobacter, Aestuariicella,* and *Alcanivorax* were detected at higher relative abundance at 0.1 MPa and 20°C. To our knowledge, this is the first study to report the presence of *Aestuariicella* in deep‐sea sediments and to show that it is negatively affected by high hydrostatic P. Available data suggest that *Halomonas* may play a greater role on hydrocarbon degradation in shallow water ecosystems even though it can survive in deep‐sea environments. Previous studies have reported a synchronous negative effect of deep‐sea conditions of high P and low T on the growth of *Halomonas* isolates (Kaye & Baross, 2004). *Halomonas* also represented 5%–9% of taxa in enrichment cultures from continental shelf sediments in the FSC but was absent from deepwater libraries (Gontikaki, Potts, Anderson, & Witte, 2018; Potts et al., 2018). Despite being previously encountered in cold, oil‐contaminated marine environments (Deppe, Richnow, Michaelis, & Antranikian, 2005; Gerdes, Brinkmeyer, Dieckmann, & Helmke, 2005), *Marinobacter* was encountered in higher relative abundance at 0.1 MPa and 20°C in this study. Previous investigations using the same model oil as here have also shown the proliferation of *Marinobacter* at 20°C than at lower temperatures in FSC sediments (Potts et al., 2018), while increased P (35 MPa) did not affect the growth rate or capability for hexadecane degradation of *Marinobacter hydrocarbonoclasticus* within 13 days (Grossi et al., 2010). *Alcanivorax* was not represented in the 20 most abundant taxa in the Hatton–Rockall Basin deep‐sea sediments in this study. *Alcanivorax* dominated an enriched HDB community from continental shelf sediments of the FSC but was absent from deepwater sediments (500–1,000 m) in the same area (Gontikaki et al., 2018; Potts et al., 2018). This study confirms the piezosensitivity of *Alcanivorax* that may explain its absence from deepwater hydrocarbon‐degrading microbial communities following the *DwH* oil spill (Kimes et al., 2013; Scoma et al., 2016). *Fusibacter* (Phylum Firmicutes) was found in higher relative abundance in 20°C, 0.1 MPa, and oil‐contaminated incubations. *Fusibacter* has been previously described as a sulfur reducer at mesophilic Ts (Serrano et al., 2017). Type strain *Fusibacter paucivorans* has been isolated from an oil well (Ravot et al., 1999) and *Fusibacter bizertensis* from a kerosene tank (Smii et al., 2015). *Fusibacter* has also been detected in hydrocarbon exposed 1,000‐m‐deep sediments from the FSC (Perez Calderon et al., 2018), which supports its ability to survive in high P environments.

Bacterial taxa that responded positively to high pressure included the γ‐proteobacteria *Pseudomonas* and *Colwellia*, as well as several Bacteroidetes. *Pseudomonas* is often associated with the early stages of oil biodegradation in both coastal and deepwater ecosystems (Dubinsky et al., 2013; Kostka et al., 2011; Krolicka, Boccadoro, Nilsen, & Baussant, 2017; Perez Calderon et al., 2018) but was found in higher relative abundance in the oil‐contaminated treatment even after 6 months of incubation in this study. The presence of *Pseudomonas* has also been reported after one‐year incubations of melted Arctic sea ice contaminated with oil (Gerdes et al., 2005). *Colwellia* also increased in relative abundance at 30 MPa but was associated with control incubations while the effect of temperature was unclear; *Colwellia* increased at 20°C, but unclassified OTUs of the Colwelliaceae family increased in relative abundance at 5°C. *Colwellia* was identified as one of the key responders in the *DwH* oil spill (Mason, et al., 2014a, Mason, et al., 2014b); however, the presence of this genus in both oil‐contaminated and uncontaminated natural bacterial communities reflects its heterotrophic nature and variable contribution to organic matter degradation (Ferguson et al., 2017; Krolicka et al., 2017; Perez Calderon et al., 2018). Within the Bacteroidetes phylum, the genera *Aequorivita*,* Flavobacterium,* and *Tenacibaculum* were found in higher relative abundance at 30 MPa. Proliferation of Bacteroidetes induced by high P has been previously reported in coastal seawater samples showing capability of this phylum to adapt to hydrostatic P changes (Fasca et al., 2017; Marietou & Bartlett, 2014). The Bacteroidetes phylum appears to play an important role in structuring bacterial communities in oil‐contaminated marine environments (Jiménez, Viñas, Bayona, Albaiges, & Solanas, 2007; Kasai, Kishira, Syutsubo, & Harayama, 2001; Koo, Mojib, Thacker, & Bej, 2014) although present knowledge of the specific role of Bacteroidetes in hydrocarbon degradation in marine environments is limited; *Aequorivita* has been previously associated with PAH biodegradation; and a *Tenacibaculum* strain isolated from coastal seawater was capable of crude oil degradation (Wang, Zhong, Shan, & Shao, 2014). In this study, oil contamination decreased the relative abundance of Bacteroidetes.

The present study presents valuable data on the synergistic effect of P, T, and oil contamination on the structure and function of the microbial community in deep‐sea sediments from the Hatton–Rockall Basin. The difficulty in high‐pressure experimentation is well known, and experimental datasets on oil degradation in deep sediments are rare. In this study, limitations posed by the number of pressure chambers and treatment combinations meant that only one end‐point could be considered. The 6‐month incubation period was largely based on previous studies in the Gulf of Mexico which revealed changes in the structure of undisturbed deepwater sediment communities 4–5 months following the *DwH* oil spill (Kimes et al., 2013; Mason, et al., 2014a, Mason, et al., 2014b). It is acknowledged that the long incubation period employed here is likely to have resulted in nutrient limitation with unknown consequences for bacterial growth and function. Nevertheless, Gerdes et al. (2005) reported that a significant fraction of Arctic sea‐ice bacteria were active or growing in the presence of crude oil after one year of incubation without intervention. The relatively long decompression phase prior to repressurization in this study could have also negatively impacted the piezophilic fraction of the bacterial community. Whether obligate barophilic bacteria constituted a large fraction of the HDB community or significantly affected the rate of hydrocarbon degradation cannot be evaluated at this stage. No interruption of pressure conditions between sampling and experimentation would be preferable in this case, but, while this has been achieved successfully for water column samples (Tamburini et al., 2002), it remains challenging for sediments (Jackson et al., 2017).

## CONFLICT OF INTEREST

The authors declare no competing interests.

## AUTHORS CONTRIBUTION

LJP, EG, LP, UW, and JA conceived the study. UW collected the samples and provided pressure incubation equipment. LJP, EG, LP, and JA conducted the experiment. SS carried out the bioinformatics analysis. LJP carried out the hydrocarbon and analysis sediment characterization. SS and LJP carried out the statistical analysis. LJP and EG wrote the paper with input from all co‐authors.

## ETHICS STATEMENT

None required.

## Data Availability

The raw sequencing data are available in the European Nucleotide Archive (ENA) under the accession number PRJEB25365.
