# Supplementary material for: Pressure and temperature effects on deep‐sea hydrocarbon‐degrading microbial communities in subarctic sediments
Source: Microbiologyopen. 2018 Nov 16;8(6):e00768. doi: 10.1002/mbo3.768 (PMC6562134; doi:10.1002/mbo3.768)
Supplement: Supplementary file 1 [file MBO3-8-e00768-s001.docx]

Pressure and temperature effects on deep-sea hydrocarbon-degrading microbial communities in subarctic sediments – Supplementary material

Luis J. Perez Calderon^1,2,3,5*^, Evangelia Gontikaki^1,5^, Lloyd D. Potts^1,2^, Sophie Shaw^4^, Alejandro Gallego^3^, James A. Anderson^2^, Ursula Witte^1^

^1^Institute of Biological and Environmental Science, University of Aberdeen, Aberdeen, United Kingdom

^2^Surface Chemistry and Catalysis Group, Materials and Chemical Engineering, School of Engineering, University of Aberdeen, Aberdeen, United Kingdom

^3^Marine Scotland Science, Marine Laboratory Aberdeen, Aberdeen, United Kingdom

^4^Centre for Genome Enabled Biology and Medicine, University of Aberdeen, Aberdeen, United Kingdom

^5^These authors contributed equally to the study.

*Corresponding author: Luis Perez Calderon (lj.perezcalderon@gmail.com)

# Supplementary figures


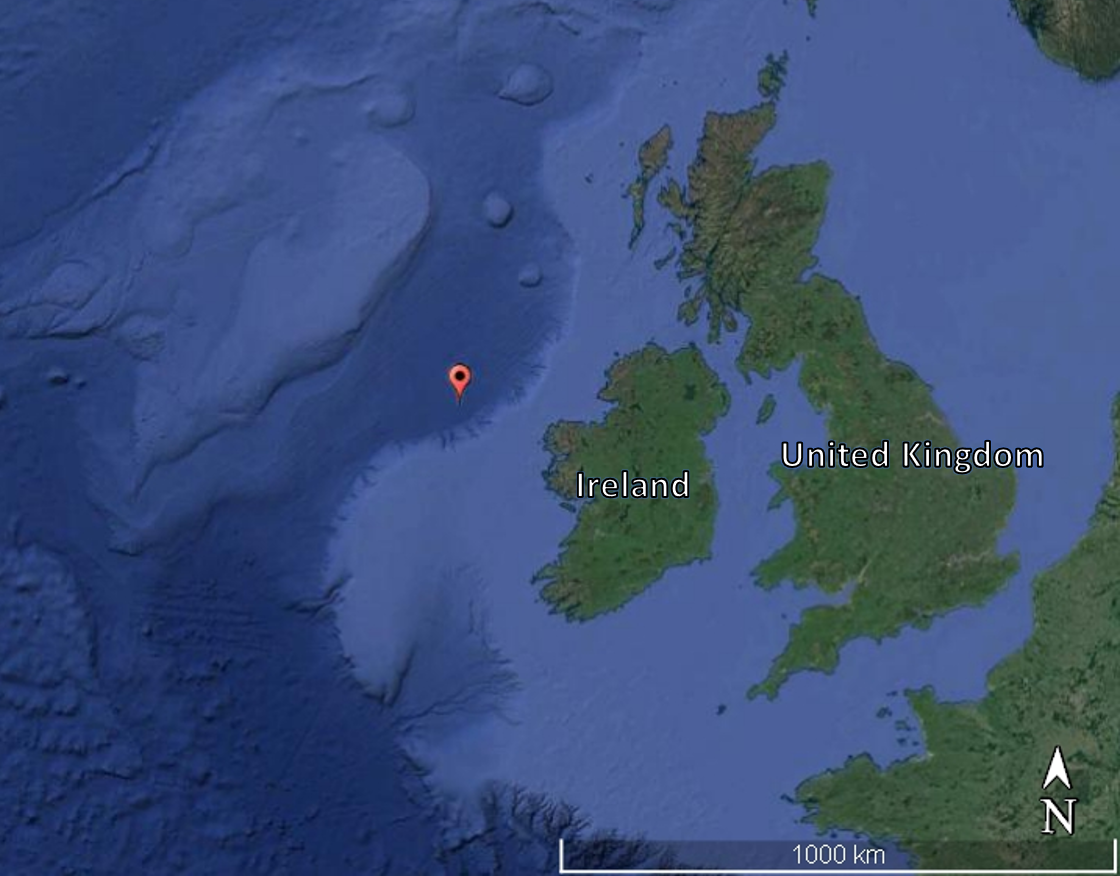


Supplementary Figure 1. Location of sampling station of this study.

Supplementary Figure 2. Rarefaction curves of all samples using observed species data.

Supplementary Figure 3. Relative abundance of taxa at the phylum level stratified by treatment, pressure and temperature.


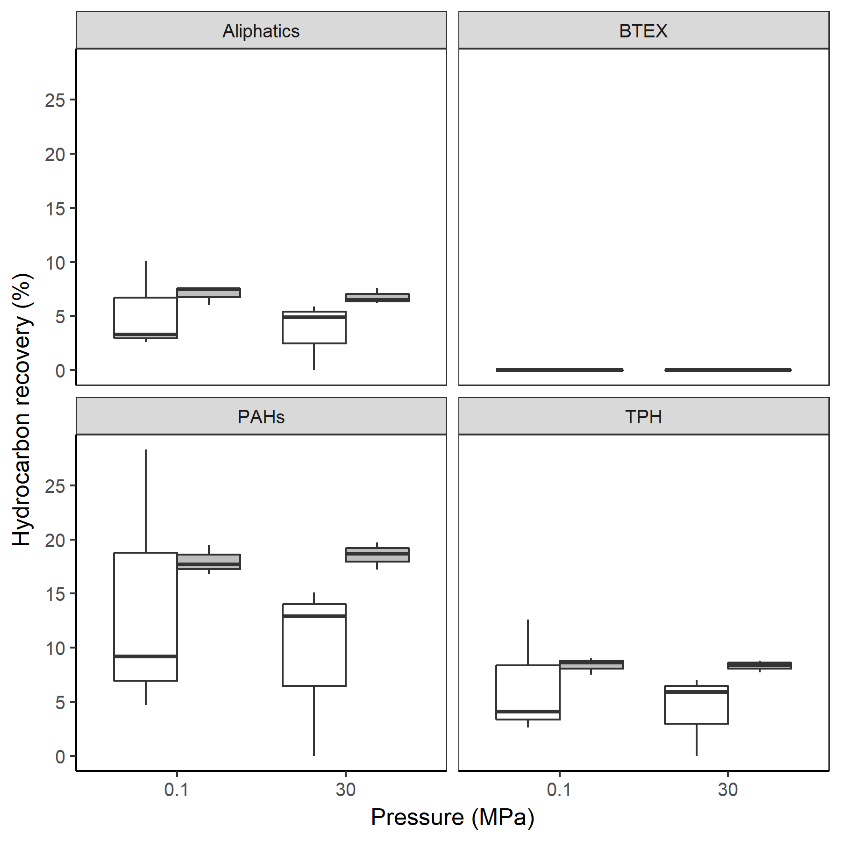


Supplementary Figure 4. Hydrocarbon recovery grouped by fraction after sediment incubation experiments. 5 (white) and 20°C (grey) incubations are shown as box plots (*n* = 3).


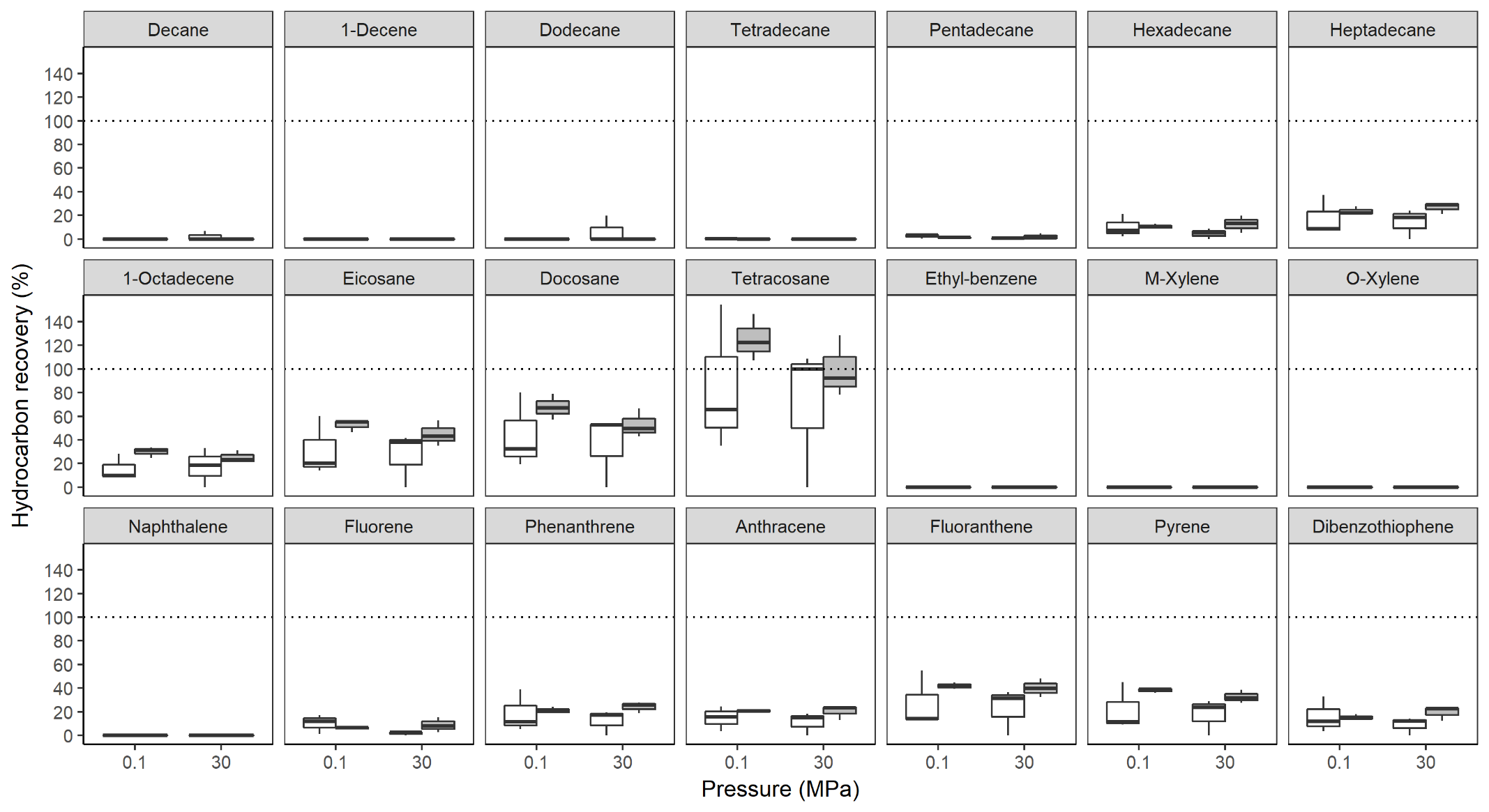


Supplementary Figure 5. Single component hydrocarbon recovery after sediment incubation experiments. 5 (white) and 20°C (grey) incubations are shown as box plots (*n* = 3). The dotted line represents 100% recovery.

# Supplementary tables

Supplementary Table 1. List of model oil components used in sediment incubation experiments (Perez Calderon *et al.*, under review).

| **Hydrocarbon** | **Hydrocarbon class** | **Mass composition (%)** |
| --- | --- | --- |
| Ethyl-Benzene | BTEX (17.6%) | 5.9 |
| m-Xylene |  | 5.9 |
| o-Xylene |  | 5.9 |
| Decane | Aliphatics (59.2%) | 8.9 |
| 1-Decene |  | 9.0 |
| Dodecane |  | 9.1 |
| Tetradecane |  | 9.4 |
| Pentadecane |  | 6.3 |
| Hexadecane |  | 8.9 |
| Heptadecane |  | 1.5 |
| 1-Octadecene |  | 1.4 |
| Eicosane |  | 1.6 |
| Docosane |  | 1.5 |
| Tetracosane |  | 1.6 |
| Naphthalene | PAHs (18.3%) | 5.4 |
| Fluorene |  | 3.1 |
| Phenanthrene |  | 2.9 |
| Anthracene |  | 0.7 |
| Fluoranthene |  | 3.0 |
| Pyrene |  | 3.2 |
| Dibenzothiophene | PAH/Resin (4.9%) | 4.9 |

Supplementary Table 2. Sediment properties of Rockall sediments used in the experiments. Errors represent standard deviation (*n* = 4). TC, TIC and TOC stand for Total Carbon, Total Inorganic Carbon and Total Organic Carbon.

| **Sand (%)** | **Silt (%)** | **Clay (%)** | **TC (%)** | **TIC (%)** | **TOC (%)** |
| --- | --- | --- | --- | --- | --- |
| 9.5 ± 1.3 | 83.0 ± 2.2 | 7.5 ± 1.0 | 1.71 ± 0.01 | 1.24 ± 0.22 | 0.47 ± 0.23 |

Supplementary Table 3. Shannon index model summary of sediment incubation experiments. Degrees of freedom are abbreviated as D.F.

|  | **D.F.** | **Sum Sq.** | **Mean Sq.** | **F** | **p** |
| --- | --- | --- | --- | --- | --- |
| Treatment | 1 | 1.044 | 1.044 | 3.496 | 0.0799 |
| Temperature | 1 | 5.479 | 5.479 | 18.352 | 0.0006 |
| Pressure | 1 | 5.522 | 5.522 | 18.495 | 0.0006 |
| Treatment × Temperature | 1 | 4.322 | 4.322 | 14.476 | 0.0016 |
| Temperature × Pressure | 1 | 5.113 | 5.113 | 17.124 | 0.0007 |
| Residuals | 16 | 4.777 | 0.299 |  |  |

Supplementary Table 4. Analysis of variance model summary of relative abundance at the family level in sediment incubation experiments.

| **Family** | **Parameter** | **D.F.** | **Sum Sq.** | **Mean Sq.** | **F** | **p** |
| --- | --- | --- | --- | --- | --- | --- |
| Alcanivoracaceae | Treatment | 1 | 0.0002 | 0.0002 | 19.1200 | 0.0003 |
|  | Residuals | 20 | 0.0002 | 0.0000 |  |  |
| Alteromonadaceae | Treatment | 1 | 0.0089 | 0.0089 | 10.0410 | 0.0053 |
|  | Pressure | 1 | 0.0175 | 0.0175 | 19.7790 | 0.0003 |
|  | Treatment × Pressure | 1 | 0.0087 | 0.0087 | 9.7980 | 0.0058 |
|  | Residuals | 18 | 0.0159 | 0.0009 |  |  |
| Flavobacteriaceae | Treatment | 1 | 0.0282 | 0.0282 | 27.5160 | 0.0001 |
|  | Pressure | 1 | 0.0238 | 0.0238 | 23.1780 | 0.0002 |
|  | Temperature | 1 | 0.0119 | 0.0119 | 11.6540 | 0.0033 |
|  | Treatment × Pressure | 1 | 0.0051 | 0.0051 | 4.9360 | 0.0402 |
|  | Residuals | 17 | 0.0174 | 0.0010 |  |  |
| Halomonadaceae | Pressure | 1 | 0.0688 | 0.0688 | 9.8860 | 0.0053 |
|  | Temperature | 1 | 0.0623 | 0.0623 | 8.9390 | 0.0075 |
|  | Residuals | 19 | 0.1323 | 0.0070 |  |  |
| Pseudomonadaceae | Treatment | 1 | 0.1310 | 0.1310 | 12.9500 | 0.0018 |
|  | Residuals | 20 | 0.2024 | 0.0101 |  |  |
| Rhodobacteraceae | Treatment | 1 | 0.0218 | 0.0218 | 18.3190 | 0.0006 |
|  | Pressure | 1 | 0.0001 | 0.0001 | 0.0810 | 0.7795 |
|  | Temperature | 1 | 0.0074 | 0.0074 | 6.2280 | 0.0239 |
|  | Treatment × Temperature | 1 | 0.0075 | 0.0075 | 6.3140 | 0.0231 |
|  | Pressure × Temperature | 1 | 0.0057 | 0.0057 | 4.7980 | 0.0436 |
|  | Residuals | 16 | 0.0191 | 0.0012 |  |  |

Supplementary Table 5. Average relative abundance of operating taxonomic units (at 97% similarity) of interest which expressed a significant change in differential abundance testing (adjusted p <0.05) with temperatures of 5 and 20 °C. Letters assigned to genera refer to separate operational taxonomic units within each genus.

| **Family** | **Genus** | **5 °C** | **20 °C** | **Adj. p** |
| --- | --- | --- | --- | --- |
| Alcanivoracaceae | *Alcanivorax* | 9.22 x 10^-5^ | 7.97 x 10^-4^ | 0.0266 |
| Alteromonadaceae | *Marinobacter_a* | 6.84 x 10^-4^ | 4.31 x 10^-3^ | 0.0161 |
|  | *Marinobacter_b* | 0 | 4.49 x 10^-3^ | 0.0000 |
|  | *Marinobacter_c* | 0 | 4.22 x 10^-3^ | 0.0000 |
|  | *Marinobacter_d* | 1.54 x 10^-5^ | 3.44 x 10^-3^ | 0.0000 |
|  | *Marinobacter_e* | 0 | 2.09 x 10^-3^ | 0.0000 |
|  | *Marinobacter_f* | 0 | 1.77 x 10^-3^ | 0.0000 |
|  | *Marinobacter_g* | 0 | 3.36 x 10^-4^ | 0.0277 |
| Cellvibrionaceae | *Aestuariicella* | 0 | 7.71 x 10^-3^ | 0.0000 |
| Clostridiales unc. | *Fusibacter_a* | 6.76 x 10^-4^ | 4.74 x 10^-3^ | 0.0221 |
| Colwelliaceae | *Colwellia* | 0 | 1.84 x 10^-3^ | 0.0000 |
|  | *Unclassified_a* | 1.35 x 10^-3^ | 2.55 x 10^-5^ | 0.0011 |
|  | *Unclassified_b* | 8.83 x 10^-4^ | 3.39 x 10^-5^ | 0.0217 |
|  | *Thalassomonas* | 6.39 x 10^-3^ | 0 | 0.0002 |
| Flammeovirgaceae | *Fulvivirga* | 1.76 x 10^-3^ | 4.25 x 10^-5^ | 0.0049 |
| Flavobacteriaceae | *Aequorivita* | 1.12e-02 | 0 | 0.0002 |
|  | *Aquibacter_a* | 1.01e-02 | 7.85 x 10^-4^ | 0.0002 |
|  | *Aquibacter_b* | 3.01 x 10^-3^ | 9.63 x 10^-4^ | 0.0282 |
|  | *Aquibacter_c* | 8.10 x 10^-4^ | 0 | 0.0108 |
|  | *Flavobacterium* | 7.78 x 10^-4^ | 0 | 0.0296 |
|  | *Tenacibaculum* | 8.68 x 10^-3^ | 4.22 x 10^-5^ | 0.0001 |
| Halomonadaceae | *Halomona_a* | 1.92 x 10^-4^ | 2.34 x 10^-3^ | 0.0007 |
|  | *Halomonas_b* | 0 | 4.12 x 10^-4^ | 0.0199 |
| Idiomarinaceae | *Idiomarina_a* | 2.54 x 10^-4^ | 1.73 x 10^-2^ | 0.0000 |
|  | *Idiomarina_b* | 1.54 x 10^-5^ | 6.00 x 10^-3^ | 0.0000 |
| Moritellaceae | *Moritella* | 3.08 x 10^-3^ | 5.32 x 10^-4^ | 0.0162 |
| Piscirickettsiaceae | *Cycloclasticus_a* | 1.95 x 10^-3^ | 0 | 0.0053 |
| Pseudomonadaceae | *Pseudomonas_a* | 7.62 x 10^-6^ | 4.57 x 10^-2^ | 0.0000 |
|  | *Pseudomonas_b* | 1.89 x 10^-2^ | 5.93 x 10^-5^ | 0.0001 |
|  | *Pseudomonas_c* | 9.94 x 10^-3^ | 0 | 0.0002 |
|  | *Pseudomonas_d* | 6.70 x 10^-3^ | 1.85 x 10^-4^ | 0.0002 |
|  | *Pseudomonas_e* | 0 | 1.05 x 10^-3^ | 0.0001 |
| Rhodobacteraceae | *Sulfitobacter_a* | 6.50 x 10^-3^ | 4.10 x 10^-2^ | 0.0162 |
|  | *Sulfitobacter_b* | 5.23 x 10^-4^ | 6.77 x 10^-3^ | 0.0001 |
| Shewanellaceae | *Shewanella_a* | 5.59 x 10^-3^ | 2.72 x 10^-4^ | 0.0002 |
|  | *Shewanella_b* | 1.14 x 10^-2^ | 2.79 x 10^-4^ | 0.0007 |
|  | *Shewanella_c* | 2.98 x 10^-3^ | 0 | 0.0007 |
|  | *Shewanella_d* | 2.22 x 10^-3^ | 8.45 X 10^-6^ | 0.0033 |
|  | *Shewanella_e* | 7.69 x 10^-5^ | 1.06 X 10^-3^ | 0.0045 |

Supplementary Table 6. Average relative abundance of operating taxonomic units (at 97% similarity) of interest which expressed a significant change in differential abundance testing (adjusted p <0.05) with pressure. Letters assigned to genera refer to separate operational taxonomic units within each genus.

| **Family** | **Genus** | **0.1 MPa** | **30 MPa** | **Adj. p** |
| --- | --- | --- | --- | --- |
| Alcanivoracaceae | *Alcanivorax* | 7.93 x 10^-4^ | 2.31 x 10^-5^ | 0.0050 |
| Alteromonadaceae | *Marinobacter_c* | 4.08 x 10^-3^ | 0 | 0.0004 |
|  | *Marinobacter_d* | 3.09 x 10^-3^ | 4.59 x 10^-5^ | 0.0005 |
|  | *Marinobacter_e* | 1.88 x 10^-3^ | 2.30 x 10^-5^ | 0.0023 |
|  | *Marinobacter_f* | 1.60 x 10^-3^ | 7.65 x 10^-6^ | 0.0012 |
|  | *Marinobacter_h* | 4.01 x 10^-2^ | 2.78 x 10^-3^ | 0.0035 |
|  | *Marinobacter_i* | 4.87 x 10^-3^ | 4.76 x 10^-4^ | 0.0155 |
| Cellvibrionaceae | *Aestuariicella* | 7.01 x 10^-3^ | 0 | 0.0002 |
| Clostridiales unc. | *Fusibacter_b* | 6.79 x 10^-3^ | 7.86 x 10^-4^ | 0.0141 |
| Colwelliaceae | *Colwellia* | 1.08 x 10^-4^ | 8.13 x 10^-4^ | 0.0202 |
|  | *Thalassomonas* | 1.62 x 10^-4^ | 6.23 x 10^-3^ | 0.0126 |
| Flammeovirgaceae | *Fulvivirga* | 5.38 x 10^-5^ | 1.75 x 10^-3^ | 0.0050 |
| Flavobacteriaceae | *Aequorivita* | 7.73 x 10^-6^ | 1.12 x 10^-2^ | 0.0003 |
|  | *Aquibacter_d* | 1.21 x 10^-3^ | 9.96 x 10^-3^ | 0.0057 |
|  | *Aquibacter_e* | 7.69 x 10^-6^ | 8.02 x 10^-4^ | 0.0135 |
|  | *Flavobacterium* | 0 | 7.78 x 10^-4^ | 0.0266 |
|  | *Tenacibaculum* | 1.23 x 10^-4^ | 8.66 x 10^-3^ | 0.0008 |
| Halomonadaceae | *Halomonas_a* | 2.21 x 10^-3^ | 1.31 x 10^-4^ | 0.0007 |
| Idiomarinaceae | *Idiomarina_a* | 1.56 x 10^-2^ | 3.59 x 10^-4^ | 0.0019 |
|  | *Idiomarina_b* | 5.40 x 10^-3^ | 6.12 x 10^-5^ | 0.0006 |
| Oceanospirillaceae | *Amphritea* | 1.50 x 10^-2^ | 4.76 x 10^-4^ | 0.0004 |
| Piscirickettsiaceae | *Cycloclasticus_a* | 7.71 x 10^-6^ | 1.94 x 10^-3^ | 0.0050 |
|  | *Cycloclasticus_b* | 8.51 x 10^-3^ | 3.61 x 10^-4^ | 0.0006 |
|  | *Cycloclasticus_c* | 1.24 x 10^-3^ | 3.08 x 10^-5^ | 0.0040 |
| Pseudomonadaceae | *Pseudomonas_b* | 3.09 x 10^-5^ | 1.89 x 10^-2^ | 0.0000 |
|  | *Pseudomonas_c* | 7.70 x 10^-5^ | 9.86 x 10^-3^ | 0.0013 |
|  | *Pseudomonas_e* | 0 | 9.56 x 10^-4^ | 0.0012 |
|  | *Pseudomonas_f* | 1.55 x 10^-5^ | 3.66 x 10^-2^ | 0.0000 |
|  | *Pseudomonas_g* | 0 | 9.32 x 10^-4^ | 0.0023 |
| Rhodobacteraceae | *Pseudophaeobacter* | 1.06 x 10^-3^ | 1.30 x 10^-4^ | 0.0495 |
| Shewanellaceae | *Shewanella_b* | 2.38 x 10^-4^ | 1.14 x 10^-2^ | 0.0003 |
|  | *Shewanella_c* | 0 | 2.98 x 10^-3^ | 0.0005 |
|  | *Shewanella_d* | 2.22 x 10^-3^ | 7.68 x 10^-6^ | 0.0018 |
|  | *Shewanella_e* | 9.68 x 10^-4^ | 7.71 x 10^-5^ | 0.0258 |
|  | *Shewanella_f* | 6.65 x 10^-4^ | 7.67 x 10^-5^ | 0.0394 |

Supplementary Table 7. Average relative abundance of operating taxonomic units (at 97% similarity) of interest which expressed a significant change in differential abundance testing (adjusted p <0.05) with treatments of control and oil. Letters assigned to genera refer to separate operational taxonomic units within each genus.

| **Family** | **Genus** | **Control** | **Oil** | **Adj. p** |
| --- | --- | --- | --- | --- |
| Alteromonadaceae | *Marinobacter_c* | 7.54 x 10^-6^ | 4.07 x 10^-3^ | 0.0005 |
|  | *Marinobacter_e* | 2.30 x 10^-5^ | 1.88 x 10^-3^ | 0.0029 |
|  | *Marinobacter_f* | 3.03 x 10^-5^ | 1.58 x 10^-3^ | 0.0050 |
| Cellvibrionaceae | *Aestuariicella* | 0 | 7.01 x 10^-3^ | 0.0001 |
| Clostridiales unc. | *Fusibacter_a* | 4.05 x 10^-4^ | 4.58 x 10^-3^ | 0.0119 |
|  | *Fusibacter_b* | 8.19 x 10^-4^ | 6.76 x 10^-3^ | 0.0184 |
|  | *Fusibacter_c* | 6.13 x 10^-5^ | 8.17 x 10^-3^ | 0.0000 |
|  | *Fusibacter_d* | 6.07 x 10^-5^ | 1.97 x 10^-3^ | 0.0004 |
| Colwelliaceae | *Colwellia* | 8.06 x 10^-4^ | 1.15 x 10^-4^ | 0.0265 |
|  | *Thalassomonas* | 1.54 x 10^-5^ | 6.38 x 10^-3^ | 0.0004 |
|  | *Unclassified_a* | 1.30 x 10^-3^ | 7.70 x 10^-5^ | 0.0118 |
| Flammeovirgaceae | *Fulvivirga* | 1.73 x 10^-3^ | 7.70 x 10^-5^ | 0.0135 |
| Flavobacteriaceae | *Aequorivita* | 1.10 x 10^-2^ | 1.54 x 10^-4^ | 0.0070 |
|  | *Aquibacter_d* | 9.79 x 10^-3^ | 1.38 x 10^-3^ | 0.0175 |
|  | *Aquibacter_e* | 8.10 x 10^-4^ | 0 | 0.0084 |
|  | *Flavobacterium* | 7.70 x 10^-4^ | 7.70 x 10^-6^ | 0.0358 |
|  | *Tenacibaculum* | 8.68 x 10^-3^ | 1.07 x 10^-4^ | 0.0006 |
| Halomonadaceae | *Cobetia* | 1.52 x 10^-5^ | 4.37 x 10^-2^ | 0.0000 |
| Idiomarinaceae | *Idiomarina_b* | 5.26 x 10^-3^ | 2.06 x 10^-4^ | 0.0154 |
| Moritellaceae | *Moritella* | 5.53 x 10^-4^ | 3.11 x 10^-3^ | 0.0157 |
| Oceanospirillaceae | *Amphritea* | 1.45 x 10^-2^ | 9.33 x 10^-4^ | 0.0119 |
| Piscirickettsiaceae | *Cycloclasticus_a* | 7.71 x 10^-6^ | 1.94 x 10^-3^ | 0.0063 |
|  | *Cycloclasticus_c* | 1.25 x 10^-3^ | 2.31 x 10^-5^ | 0.0029 |
| Pseudomonadaceae | *Pseudomonas_a* | 9.12 x 10^-4^ | 4.06 x 10^-2^ | 0.0149 |
|  | *Pseudomonas_c* | 0 | 9.94 x 10^-3^ | 0.0001 |
|  | *Pseudomonas_h* | 3.84 x 10^-5^ | 6.52 x 10^-2^ | 0.0000 |
| Rhodobacteraceae | *Pseudophaeobacter* | 1.08 x 10^-3^ | 1.07 x 10^-4^ | 0.0184 |
|  | *Sulfitobacter_b* | 6.03 x 10^-3^ | 6.52 x 10^-4^ | 0.0119 |
| Shewanellaceae | *Shewanella_c* | 2.86 x 10^-3^ | 1.23 x 10^-4^ | 0.0262 |
|  | *Shewanella_d* | 7.68 x 10^-6^ | 2.22 x 10^-3^ | 0.0025 |
|  | *Shewanella_e* | 6.15 x 10^-5^ | 9.84 x 10^-4^ | 0.0135 |
|  | *Shewanella_f* | 6.13 x 10^-5^ | 6.80 x 10^-4^ | 0.0157 |
